# Effect of 24-month physical activity on cognitive frailty and the role of inflammation: the LIFE randomized clinical trial

**DOI:** 10.1186/s12916-018-1174-8

**Published:** 2018-10-24

**Authors:** Zuyun Liu, Fang-Chi Hsu, Andrea Trombetti, Abby C. King, Christine K. Liu, Todd M. Manini, Roger A. Fielding, Marco Pahor, Anne B. Newman, Stephen Kritchevsky, Thomas M. Gill, for the LIFE Study investigators

**Affiliations:** 1 Department of Internal Medicine, Yale School of Medicine, New Haven, CT USA; 2 Department of Biostatistical Sciences, Wake Forest University Health Sciences, Winston Salem, NC USA; 3 Division of Bone Diseases, Department of Internal Medicine Specialties, Geneva University Hospitals and Faculty of Medicine, Geneva, Switzerland; 4 Nutrition, Exercise Physiology and Sarcopenia Laboratory, Jean Mayer USDA Human Nutrition Research Center on Aging, Tufts University, Boston, MA USA; 5 Department of Health Research and Policy and the Stanford Prevention Research Center, Department of Medicine, Stanford University, School of Medicine, Stanford, CA USA; 6 Section of Geriatrics, Boston University School of Medicine, Boston, MA USA; 7 Department of Aging and Geriatric Research, University of Florida, Gainesville, FL USA; 8 Department of Epidemiology, Graduate School of Public Health, University of Pittsburgh, Pittsburgh, PA USA; 9 Sticht Center on Aging, Wake Forest School of Medicine, Winston Salem, NC USA

**Keywords:** Physical activity, Cognitive frailty, Interleukin-6, Older persons

## Abstract

**Background:**

Whether physical activity can reduce cognitive frailty—a relatively new “compound” phenotype proposed in 2013—and whether the effect of physical activity differs based on levels of inflammation are unknown. Therefore, this study aimed to evaluate the effect of physical activity on cognitive frailty and whether baseline interleukin-6 (IL-6) levels modified this effect.

**Methods:**

We used data from the Lifestyle Interventions and Independence for Elders (LIFE) Study, a multicenter, single-blinded randomized trial conducted at eight US field centers between February 2010 and December 2013. The main outcome was cognitive frailty at 24 months, expressed as an ordinal variable based on the six combinations of its two components: frailty (non-frail, pre-frail, and frail) and mild cognitive impairment (yes, no). Frailty and cognition were assessed by the Study of Osteoporotic Fractures (SOF) index and the Modified Mini-Mental State Examination (3MSE) scale, respectively. Plasma IL-6 was measured at baseline. Of the 1635 original randomized sedentary participants (70–89 years), this study included 1298 participants with data on both cognitive frailty and IL-6 assessments at baseline.

**Results:**

After adjusting for field center, sex, and baseline levels of cognitive frailty, the ordinal logistic regression model revealed that participants in the physical activity group had 21% lower odds (odds ratio, 0.79; 95% confidence interval, 0.64–0.98) of worsening cognitive frailty over 24 months than those in the health education group. The effect of physical activity on cognitive frailty did not differ according to baseline IL-6 levels (*P* for interaction = 0.919). The results did not change after additional adjustment for IL-6 subgroups and the inverse probability of remaining in the study. Comparable results were observed according to age, sex, ethnicity/race, and short physical performance battery score (*P* for interaction = 0.835, 0.536, 0.934, and 0.458, respectively).

**Conclusions:**

A 24-month structured, moderate-intensity physical activity program reduced cognitive frailty compared with a health education program in sedentary older persons, and this beneficial effect did not differ according to baseline levels of inflammatory biomarker IL-6. These findings suggest that the new cognitive frailty construct is modifiable and highlight the potential of targeting cognitive frailty for promoting healthy aging.

**Trial registration:**

Clinicaltrials.gov, NCT01072500

**Electronic supplementary material:**

The online version of this article (10.1186/s12916-018-1174-8) contains supplementary material, which is available to authorized users.

## Background

Cognitive impairment and physical frailty (hereafter referred to as frailty) are two important determinants of an array of adverse health outcomes in older persons that have typically been studied separately as if they were two independent processes. However, recent studies have challenged this traditional view and demonstrated their close interrelationship [1–4], involving chronic inflammation, hormones, nutrition, etc. [3]. Based on these results, a new conceptual construct—cognitive frailty—characterized by the simultaneous presence of both cognitive impairment and frailty, was proposed in 2013 by an international consensus group [5, 6]. Cognitive frailty is more predictive for adverse health outcomes such as functional disability [7–9], low quality of life [7], and mortality [7], than the two individual components, and has become a new target for healthy aging [10].

Many randomized clinical trials have evaluated the effect of interventions such as physical activity on preventing cognitive impairment, but the results have been conflicting [11–14]. In contrast, physical activity is deemed as a promising intervention for reducing frailty, despite limited evidence [15–17]. However, whether physical activity can reduce cognitive frailty, a relatively new “compound” phenotype, over an extended period of time is currently unknown.

From the perspective of putative mechanisms, physical activity has anti-inflammatory properties [18]. Cross-sectional [19–22] and longitudinal studies [23–25] have suggested that an increased inflammatory state plays a key role in the pathogenesis of frailty [26]. Likewise, research has shown links between inflammation and cognitive impairment [27, 28]. Recent reviews suggest that inflammation is one possible underlying pathogenetic pathway linking frailty to cognition [29, 30]. Furthermore, a recent study found that among nondemented older persons with increased inflammation, a cognitive frailty model has a significant additional predictive effect on the risk of disability than the individual components (i.e., frailty, or cognitive impairment) [31]. Therefore, it is plausible that the putative benefit of physical activity on cognitive frailty is more pronounced among older persons who have an increased inflammatory state. If this hypothesis is confirmed, our understanding of the underlying mechanism of cognitive frailty may be advanced and more aggressive programs may be targeted to subpopulations with heightened inflammation.

To address these unanswered questions, we used data from the Lifestyle Interventions and Independence for Elders (LIFE) Study, the largest and longest randomized trial evaluating the benefits of physical activity in older persons [32, 33]. The primary report of the LIFE Study demonstrated the benefit of a structured physical activity intervention on major mobility disability compared with a health education program among sedentary community-dwelling older persons [32]. The objectives of this current analysis were two–fold: first, to evaluate the effect of physical activity on cognitive frailty; and second, to determine whether inflammatory biomarkers at baseline, particularly interleukin-6 (IL-6), modified the effect of physical activity on cognitive frailty. Among several systemic biomarkers that are indicative of an inflammatory state, IL-6 is deemed as the “cytokine for gerontologists” in terms of its close relationship with aging and chronic morbidity [34, 35]. Furthermore, IL-6 demonstrates consistent associations with frailty [19, 20, 22] and cognitive impairment [27, 28] in older persons.

## Methods

### Trial design and participants

**Fig. 1:**
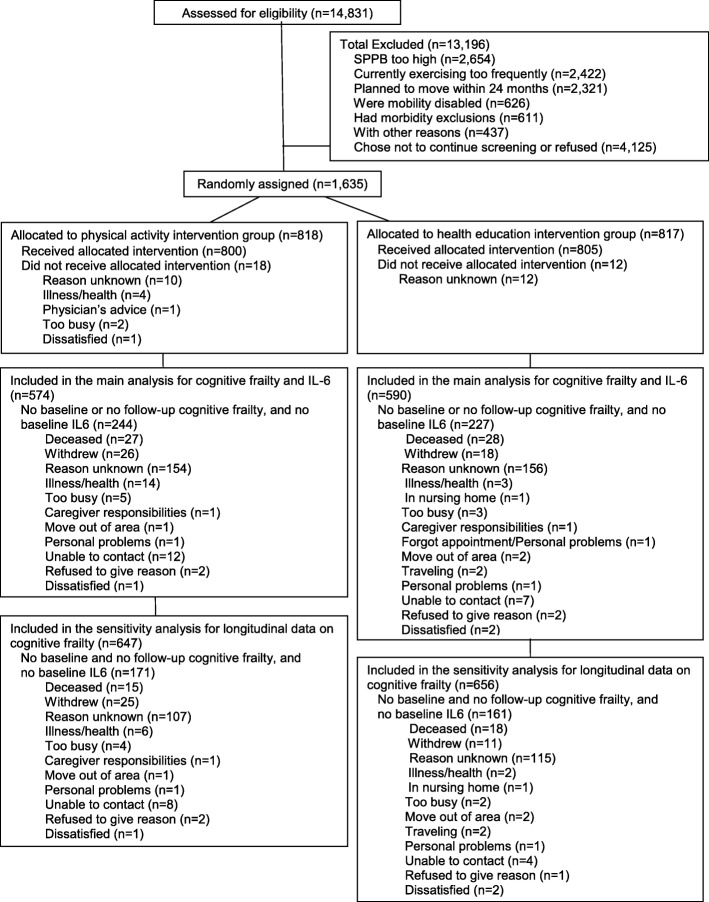
Flow of study participants through the study. SPPB, Short Physical Performance Battery; IL-6, interleukin-6. The main analysis for cognitive frailty and IL-6 indicates using the ordinal logistic regression models in which baseline levels of cognitive frailty was included as a covariate. The sensitivity analysis for longitudinal data on cognitive frailty indicates using the constrained ordinal logistic regression model in which baseline cognitive frailty was treated as an outcome

The design, recruitment, baseline characteristics, and main outcomes of the LIFE Study have been published and described in detail elsewhere [32, 33]. Briefly, the LIFE Study, a multicenter, single-blinded randomized trial (clinicaltrials.gov Identifier: NCT01072500) was conducted at eight US field centers (University of Florida, Gainesville and Jacksonville, Florida; Northwestern University, Chicago, Illinois; Pennington Biomedical Research Center, Baton Rouge, Louisiana; University of Pittsburgh, Pittsburgh, Pennsylvania; Stanford University, Stanford, California; Tufts University, Boston, Massachusetts; Wake Forest University, Winston-Salem, North Carolina; and Yale University, New Haven, Connecticut) between February 2010 and December 2013. Men and women aged 70–89 were eligible if they: (a) were sedentary (reported < 20 min/week in past month performing structured physical activity (i.e., exercise), and < 125 min/week of moderate physical activity); (b) had functional limitations, as evidenced by a short physical performance battery (SPPB) score 9 or less out of 12 (the SPPB is an integrative measure of balance, gait, and lower extremity strength); (c) could walk 400 m in 15 min or less without the help of someone or a walker; and (d) could safely participate in the intervention as determined by medical history, physical exam, and electrocardiography [32, 33]. Eligible participants had no diagnosis of dementia or significant cognitive impairment based on the Modified Mini-Mental State Examination [36] (3MSE) after accounting for education and race [32, 33]. Informed consent was obtained from all participants. The institutional review boards at all participating sites approved the study protocol. Of the 1635 original randomized participants, 1592 consented to the baseline blood draw, and a sufficient blood sample was successfully collected from 1535 (94%) participants. In this study, the 1298 participants who had baseline data on both cognitive frailty and IL-6 were included, and the main analyses focused on the subset of 1164 participants (71.2%) who also had follow-up data on cognitive frailty (Fig. 1).

### Interventions

Participants were randomly assigned via a secure web-based data management system using a permuted block algorithm (with random block lengths) to either a physical activity intervention or a health education program, stratified by field center and sex. Details about the two interventions are provided elsewhere [32, 33]. Briefly, the physical activity intervention included a goal of 150 min/week of walking, in addition to strength, flexibility, and balance training. This intervention required attendance at two center-based visits a week and home-based activity three to four times a week. The physical activity sessions were individualized and progressed towards a goal of 30 min of walking daily at moderate intensity, 10 min of primarily lower extremity strength training by means of body weight (e.g., chair rises) and ankle weights (2 sets of 10 repetitions), 3–5 min of large muscle group flexibility exercises, and 10 min of balance training. The participants began with lighter intensity and gradually increased intensity over the first 2–3 weeks of the intervention. The Borg’s scale of self-perceived exertion [37], with scores ranging from 6 to 20, was used to measure intensity of activity. Participants were asked to walk at an intensity of 13 (activity perception of “somewhat hard”), and perform lower extremity strengthening exercises at an intensity of 15 to 16 (activity perception of “hard”).

The health education group attended weekly workshops of health education during the first 26 weeks, and monthly sessions thereafter. Workshops consisted of topics that are relevant to older persons, other than physical activity, such as negotiating the healthcare system, traveling safely, preventive services, and other relevant topics. The program also included a 5- to 10-min instructor-led program of gentle upper extremity stretching or flexibility exercises.

### Measurements

#### Inflammatory biomarkers—IL-6

At the baseline visit, blood samples were collected in the early morning (between 7 and 9 a.m.) after a 12-h fast. Blood sampling was postponed (1–2 weeks after recovery of all symptoms) in the event of an acute respiratory, urinary tract, or other infections. All blood was collected, processed, divided into aliquots, and stored locally at − 80 °C until shipment to the Biological Specimen Repository at Wake Forest School of Medicine.

Plasma IL-6 was determined using the Quantikine high-sensitivity enzyme-linked immunosorbent assay kit from R&D Systems (Minneapolis, MN). All samples were measured in duplicate, and the average of the two values was used for data analyses. We categorized participants into two subgroups according to the median value (i.e., 3.31 pg/mL), as described previously [38].

### Outcomes assessment

#### SOF frailty

Frailty status was determined at baseline and 24 months using the Study of Osteoporotic Fractures (SOF) frailty index, which includes three criteria as proposed by Ensrud and colleagues [39]. Self-reported reduced energy level was based on the following question: “During the past week, how often have you felt full of energy”. The criterion was present if the participants answered, “Some of the time”, “A little bit of the time”, or “None of the time”. The second criterion, inability to rise from a chair five times without using the arms, was based on the chair rise test from the SPPB. The criterion of weight loss was based on objective measurements and considered as present if the value was ≥ 4.55 kg or ≥ 5% during the prior 12 months. Because no objective information on weight loss was available at baseline, this criterion was considered to be present at baseline if the participant answered, “some of the time”, or “most of the time” to the question, “How often in the last week did you not feel like eating because your appetite was poor”. Participants were considered non-frail if none of the three criteria was present, pre-frail if one was present, and frail if at least two were present [39].

#### Cognitive assessment

Cognition was assessed by using the 3MSE scale, which is a 100-point test of global cognitive function. As described previously [40], participants with less than 88 were considered as having mild cognitive impairment (MCI).

#### Cognitive frailty

The main outcome was cognitive frailty at 24 months. This new construct has been previously validated [7–9, 41]. To reflect its continuum [42], we created an ordinal variable based on the six combinations of the two components [7–9]: frailty (non-frail, pre-frail, and frail) and MCI (yes, no). For primary analysis, ordinal variable 1 was created by assigning 0 for no cognitive frailty (i.e., non-frail without MCI), 1 for pre-frail without MCI, 2 for frail without MCI, 3 for non-frail with MCI, 4 for pre-frail with MCI, and 5 for cognitive frailty (i.e., frail with MCI). A higher score of this ordinal variable indicates worse cognitive frailty. In the absence of a gold standard, we also created a modified version of this ordinal variable (i.e., ordinal variable 2) for secondary analysis, assigning 2 for non-frail with MCI and 3 for frail without MCI, while leaving the other values unchanged.

### Additional covariates

Information on covariates was obtained at the baseline visit, including age, sex, education, ethnicity/race, living alone, body mass index, Center for Epidemiology Studies-Depression (CES-D) scores [43], SPPB score (< 8 or 8–9), and history of hypertension, diabetes, cardiovascular disease, and stroke.

### Statistical analyses

Baseline characteristics were presented by intervention group and baseline IL-6 level using mean (standard deviation [SD]) or numbers (percentages). The distribution of cognitive frailty at baseline and 24 months were presented by intervention group.

To evaluate the effect of physical activity on cognitive frailty, an ordinal logistic regression model was used. This model is written as follows:

$$ \log\ odds\left(Y\le k\right)={\alpha}_k+{\beta}_1{X}_1+{\beta}_2{X}_2+\cdots +{\beta}_P{X}_P $$

where *k* (=1, 2, 3, 4, or 5) is the value in the outcome measure *Y* (i.e., cognitive frailty); *X*_*i*_ (*i* = 1 to *p*) is the covariate; *α*_*k*_ is the intercept respect to *k*; and *β*_*i*_ is the regression coefficient for each covariate. The covariates included intervention, field center, sex, and baseline levels of cognitive frailty (Model 1). To determine whether baseline IL-6 levels modified the effect of physical activity on cognitive frailty, we tested the interaction between intervention and IL-6 subgroups (Model 2). To account for losses to follow-up, we reran this model weighted for the inverse probability of remaining in the study (Model 3). For each participant, weights were assigned on the basis of field center, age, sex, education, ethnicity/race, living alone, SPPB score, number of chronic diseases, and 400-m gait speed [32]. Odds ratios (ORs) and their corresponding 95% confidence intervals (CIs) for the effects of intervention (physical activity vs. health education) and IL-6 subgroups (higher IL-6 vs. lower IL-6) were calculated.

Next, we performed comparisons for the four pre-specified subgroups: age (70–79 years vs. ≥ 80 years), sex (women vs. men), ethnicity/race (White vs. other), and SPPB score (< 8 vs. ≥ 8). The interactions between subgroup and intervention were included in Model 1 to determine whether the intervention effect was the same in each subgroup. In a stratified analysis, we determined the OR for the intervention effect and its 95% CIs for each subgroup.

To test the robustness of our results, we performed two sensitivity analyses. First, we ran a constrained ordinal logistic regression model incorporating generalized estimating equations, which account for the correlated longitudinal data (e.g., cognitive frailty was measured at baseline and 24 months). We adjusted for field center, sex, visit, and intervention by visit interaction. Baseline cognitive frailty was treated as an outcome, not as a covariate. Second, we reran our models using another cutoff point (2.5 pg/ml) of IL-6 as suggested in other studies [38].

In a set of secondary analyses, we repeated the above primary analyses for ordinal variable 2, and the corresponding results are presented alongside those from primary analyses. We considered two-sided *P* value < 0.05 to be statistically significant. Analyses were performed using SAS 9.4 (SAS Institute, Cary, NC).

## Results

On average, the physical activity group attended 63% of the scheduled sessions (median [interquartile range, IQR], 71% [50–83%]), while the health education group attended 73% of the scheduled sessions (median [IQR], 82% [63–90%]) [32]. Table 1 presents the baseline characteristics of participants by intervention group and baseline IL-6 level. The two intervention groups showed similar baseline characteristics. Overall, the mean age was about 79 years, two thirds were women, over 60% had education beyond high school, about three quarters were White, and nearly one half lived alone. For each intervention group, the two IL-6 subgroups also showed similar baseline characteristics with the following exceptions: the physical activity group had a higher percentage of hypertension in the higher IL-6 subgroup, while the health education group had a lower percentage of women and higher body mass index, and a higher percentage of diabetes in the higher IL-6 subgroup.

**Table 1:** Baseline characteristics of study participants by intervention group and baseline IL-6 level

	Physical activity			Health education		
	Higher IL-6 subgroup^a^(*n* = 321)	Lower IL-6 subgroup^a^(*n* = 323)	All *n* = 644	Higher IL-6 subgroup^a^(*n* = 331)	Lower IL-6 subgroup^a^(*n* = 323)	All *n* = 654
Age, years, mean (SD)	78.9 ± 5.3	78.2 ± 5.1	78.6 ± 5.2	79.1 ± 5.2	79.1 ± 5.3	79.1 ± 5.3
≥ 80 years	133 (41.4)	129 (39.9)	262 (40.7)	144 (43.5)	146 (45.2)	290 (44.3)
Women	203 (63.2)	217 (67.2)	420 (65.2)	204 (61.6)	232 (71.8)	436 (66.7)
Education						
≤ High school	121 (37.7)	125 (38.8)	246 (38.3)	111 (33.6)	113 (35.1)	224 (34.4)
College	136 (42.4)	121 (37.6)	257 (40.0)	136 (41.2)	129 (40.1)	265 (40.6)
Post graduate	64 (19.9)	76 (23.6)	140 (21.8)	83 (25.2)	80 (24.8)	163 (25.0)
Ethnicity/race						
White	245 (76.3)	242 (74.9)	487 (75.6)	260 (78.5)	256 (79.3)	516 (78.9)
African American	59 (18.4)	61 (18.9)	120 (18.6)	54 (16.3)	39 (12.1)	93 (14.2)
others	17 (5.3)	20 (6.2)	37 (5.7)	17 (5.1)	28 (8.7)	45 (6.9)
Living alone	140 (43.6)	148 (45.8)	288 (44.7)	172 (52.0)	153 (47.4)	325 (49.7)
CES-D score, mean (SD)	8.0 ± 7.0	8.4 ± 8.0	8.2 ± 7.5	8.9 ± 8.0	8.7 ± 7.9	8.8 ± 7.9
Body mass index, kg/m^2^, mean (SD)	31.0 ± 6.0	29.4 ± 5.4	30.2 ± 5.7	31.8 ± 6.4	28.8 ± 5.4	30.3 ± 6.1
SPPB score, mean (SD)	7.4 ± 1.7	7.5 ± 1.5	7.4 ± 1.6	7.2 ± 1.6	7.4 ± 1.6	7.3 ± 1.6
SPPB = 8 or 9	174 (54.2)	192 (59.4)	366 (56.8)	176 (53.2)	181 (56.0)	357 (54.6)
History of hypertension	243 (75.7)	215 (66.6)	458 (71.1)	246 (74.3)	222 (68.7)	468 (71.6)
History of diabetes	86 (26.8)	77 (23.8)	163 (25.3)	99 (29.9)	70 (21.7)	169 (25.8)
History of cardiovascular disease	27 (8.4)	17 (5.3)	44 (6.8)	32 (9.7)	19 (5.9)	51 (7.8)
History of stroke	29 (9.0)	21 (6.5)	50 (7.8)	17 (5.1)	25 (7.7)	42 (6.4)

Values are numbers (percentages) unless stated otherwise. The 1298 participants who had baseline data on both cognitive frailty and IL-6 were included

*SD* standard deviation, *CES-D* Center for Epidemiologic Studies-Depression scale, *SPPB* Short Physical Performance Battery, *IL-6* interleukin-6

^a^The cutoff point for categorizing IL-6 subgroups was 3.31 pg/mL (median value) in both physical activity and health education groups

**Fig. 2:**
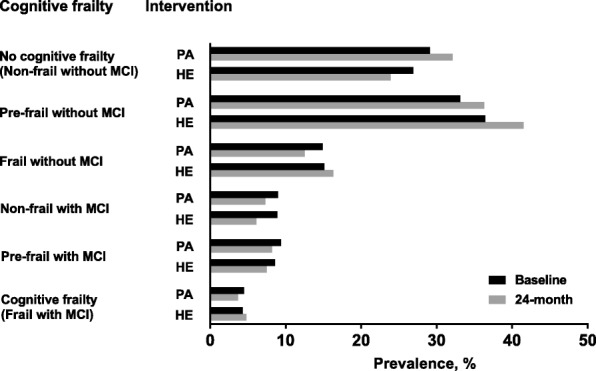
Distribution of cognitive frailty at baseline and 24 months by intervention group. PA, physical activity; HE, health education. To reflect the continuum of cognitive frailty, we created an ordinal variable based on the six combinations of two components: frailty (non-frail, pre-frail, and frail) and MCI (yes, no): no cognitive frailty (i.e., non-frail without MCI), pre-frail without MCI, frail without MCI, non-frail with MCI, pre-frail with MCI, and cognitive frailty (i.e., frail with MCI)

Figure 2 shows the distribution of cognitive frailty at baseline and 24 months by intervention group. In the physical activity group, the prevalence of non-frail without MCI and pre-frail without MCI increased from baseline to 24 months, while the prevalence of the other cognitive frailty groups decreased. In the health education group, the prevalence of pre-frail without MCI, frail without MCI, and frail with MCI increased, whereas the prevalence of non-frail without MCI, non-frail with MCI, and pre-frail with MCI decreased.

**Fig. 3:**
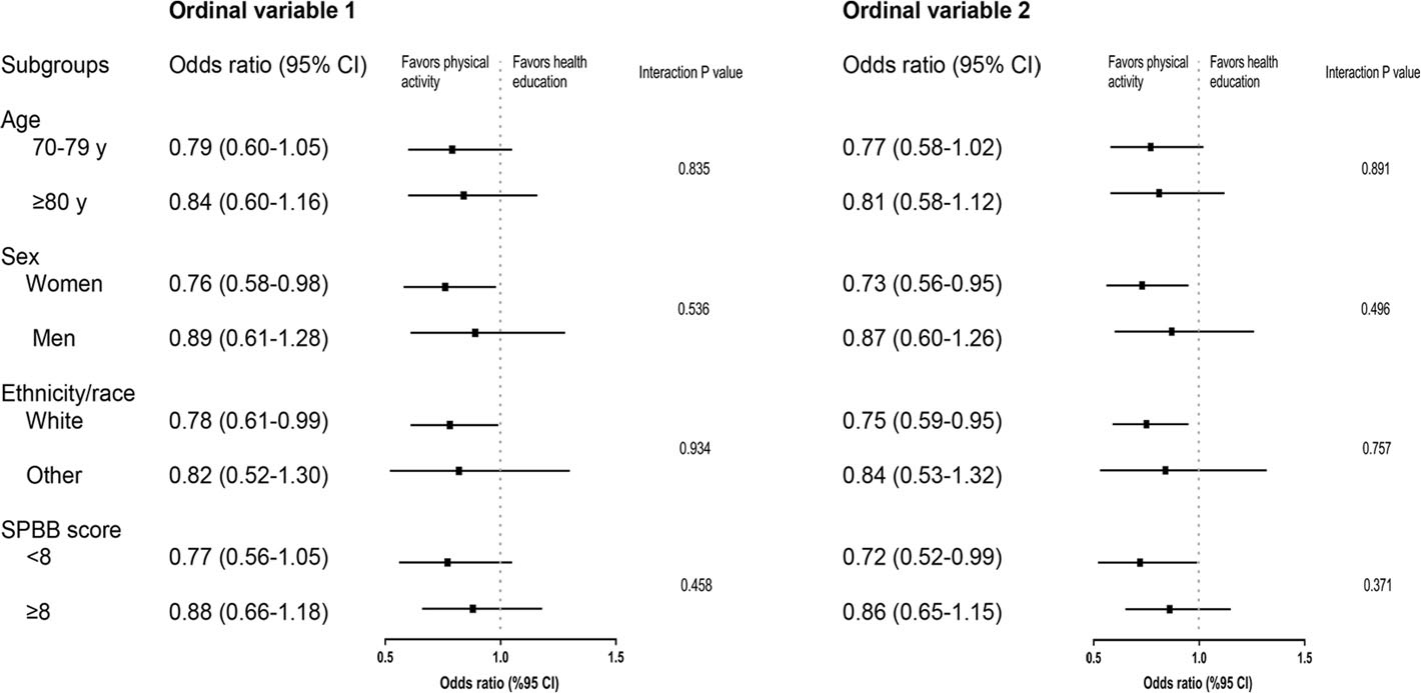
Effect of physical activity on cognitive frailty according to prespecified subgroups. CI, confidence interval. SPPB, Short Physical Performance Battery. Ordinal logistic regression was used as described in the Method section. For the primary analysis, ordinal variable 1 was created by assigning 0 for no cognitive frailty (i.e., non-frail without mild cognitive impairment [MCI]), 1 for pre-frail without MCI, 2 for frail without MCI, 3 for non-frail with MCI, 4 for pre-frail with MCI, and 5 for cognitive frailty (i.e., frail with MCI). For the secondary analysis, ordinal variable 2 was created by assigning 2 for non-frail with MCI and 3 for frail without MCI while the other values remained unchanged. The models were adjusted for intervention, field center, sex, and baseline levels for cognitive frailty. The interaction between subgroup and intervention were included to determine whether the intervention effect was the same in each subgroup. In a stratified analysis, we determined the odds ratio for the intervention effect and its 95% confidence intervals (CIs) for each subgroup

Table 2 presents the effect of physical activity on cognitive frailty. In the primary and secondary analyses, participants in the physical activity group had lower odds of worsening cognitive frailty over 24 months than those in the health education group. For example, in the primary analysis, the odds of worsening cognitive frailty was 21% (OR, 0.79; 95% CI, 0.64–0.98) lower in the physical activity group compared with the health education group. The effect of physical activity on cognitive frailty did not differ according to baseline IL-6 levels (*P* for interaction = 0.919 for primary analysis and 0.936 for secondary analysis). The results did not change after additional adjustment for IL-6 subgroups and the inverse probability of remaining in the study (Models 2 and 3). Comparable results were observed according to age, sex, ethnicity/race, or SPPB score (all *P* for interaction > 0.05, Fig. 3).

**Table 2:** Effect of physical activity on cognitive frailty using ordinal logistic regression models

	Model 1		Model 2		Model 3	
	OR (95% CI)	*P* value	OR (95% CI)	*P* value	OR (95% CI)	*P* value
Primary analysis						
Physical activity vs. health education	0.79 (0.64–0.98)	0.032	0.79 (0.64–0.98)	0.030	0.80 (0.65–0.97)	0.026
Higher IL-6 vs. lower IL-6	–	–	1.11 (0.89–1.37)	0.357	1.09 (0.89–1.33)	0.400
Secondary analysis						
Physical activity vs. health education	0.77 (0.62–0.95)	0.015	0.77 (0.62–0.95)	0.014	0.77 (0.63–0.95)	0.012
Higher IL-6 vs. lower IL-6	–	–	1.13 (0.91–1.40)	0.260	1.11 (0.91–1.36)	0.301

The 1164 participants who had baseline data on cognitive frailty and IL-6 and follow-up data on cognitive frailty were included

*OR* odds ratio, *CI* confidence interval, *IL-6* interleukin-6

Ordinal logistic regression was used as described in the Method section. For the primary analysis, ordinal variable 1 was created by assigning 0 for no cognitive frailty (i.e., non-frail without mild cognitive impairment [MCI]), 1 for pre-frail without MCI, 2 for frail without MCI, 3 for non-frail with MCI, 4 for pre-frail with MCI, and 5 for cognitive frailty (i.e., frail with MCI). For the secondary analysis, ordinal variable 2 was created by assigning 2 for non-frail with MCI and 3 for frail without MCI while the other values remained unchanged

Model 1 adjusted for intervention, field center, sex, and baseline levels for cognitive frailty

As the interaction between intervention groups and IL-6 subgroups was not statistically significant (*P* for interaction = 0.919 for primary analysis and 0.936 for secondary analysis), it was not included in Model 2

Model 3 adjusted for the same covariates as in Model 2 but weighted for the inverse probability of remaining in the study

In sensitivity analyses, we found (1) a significant intervention effect at 24 months for both versions of the cognitive frailty variable in the constrained models (Additional file 1: Table S1), for example, the odds of having worsening cognitive frailty score over 24 months was 20% (e.g., primary analysis for ordinal variable 1, OR, 0.80; 95% CI, 0.65–0.98, Model 1) lower in the physical activity group than the health education group; (2) when the alternative cutoff point was used, baseline IL-6 levels did not modify the effect of physical activity on cognitive frailty.

## Discussion

To the best of our knowledge, this is the first study to evaluate the effect of long-term physical activity on cognitive frailty, a new construct in older persons [5]. This study demonstrated that a 24-month structured, moderate-intensity physical activity program reduced the severity of cognitive frailty compared with a health education program among sedentary older persons and that this benefit was not modified by baseline IL-6.

A recent systematic review with meta-analysis concluded that physical exercise, including moderate intensity aerobic and resistance training, could improve cognitive function in persons aged 50 years or older, regardless of baseline cognitive status [44]. Another review suggested that multicomponent exercises, including aerobic, resistance, balance, and/or flexibility training, have the most positive effects on cognitive function in healthy older persons over 65 years old [11]. These results are further supported by those described in a systematic review of 32 trials [14]. However, prior results from the LIFE Study showed no difference in global cognition between the two intervention groups [12]. In contrast, interventions such as physical activity have demonstrated relatively consistent positive effects on reversing frailty and/or its components [15–17, 45]. For example, a recent secondary analysis from the LIFE Study found that the 24-month physical activity program was associated with improvement in one criterion (i.e., the inability to rise from a chair five times) of the SOF frailty index [17]. In a recent trial, a 1-year multicomponent exercise intervention including physical activity diminished frailty and improved cognition in frail community-dwelling older persons [46]. Building on these prior results [11–17, 44–46], we provide the first clinical trial evidence for the beneficial effect of physical activity on cognitive frailty in sedentary older persons, suggesting that cognitive frailty may be modifiable and has the potential to serve as a target for promoting healthy aging [10].

We found that baseline IL-6 levels did not modify the beneficial effect of physical activity on cognitive frailty, indicating that the effect of intervention was not influenced by underlying inflammation. This result suggests that inflammation may not be an important pathophysiological factor of cognitive frailty, despite prior beliefs established on the basis of evidence on cognition and/or frailty [29, 30, 47]. Alternatively, IL-6 may not be the best indicator of inflammation in older persons with functional limitations [48], although it has been widely used in previous studies [34]. Future studies should consider evaluating multiple biomarkers (e.g., tumor necrosis factor-α) to assess the underlying inflammatory state [49].

The current study has many strengths, including the large sample of an older vulnerable population who were typically excluded in prior randomized trials of physical activity, long duration of intervention and follow-up period, and high retention rate. The availability of IL-6 further provides a unique opportunity to examine the role of inflammation. However, this study also has several limitations. First, the exclusion of participants with dementia or significant cognitive impairment, and those without functional limitation and/or IL-6, may yield a biased sample, reducing the generalizability of the findings. Second, cognitive frailty was neither an entry criterion nor a randomization stratum since this was a secondary analysis that was not pre-specified in the original protocol. Third, because information was not available on weight loss at baseline, loss of appetite was substituted as one of the frailty criteria [50]. Fourth, although frailty and MCI may affect health to differing degrees, each of the cognitive frailty groups was weighted equally when creating the ordinal variable. Fifth, power may not have been adequate to detect a modifying effect of IL-6, which was also not pre-specified; therefore, future studies with larger sample size are warranted. Finally, the physical activity intervention included a coordinated program of endurance, strength, flexibility, and balance training, making it difficult to formally separate which components of the intervention were effective in reducing cognitive frailty.

## Conclusions

Compared with a health education program, a structured, moderate-intensity physical activity program reduced the severity of cognitive frailty over 2 years among sedentary older persons, but this beneficial effect did not differ according to the baseline levels of inflammatory biomarker IL-6. These findings suggest that the new cognitive frailty construct is modifiable and highlight the potential of targeting cognitive frailty for promoting healthy aging.

## Additional files

Additional file 1: **Table S1.** Effect of physical activity on cognitive frailty using constrained ordinal logistic regression models. (DOCX 29 kb)

Additional file 2: Research investigators for the LIFE Study. (DOCX 15 kb)

## Abbreviations

3MSE: Modified Mini-Mental State Examination
CES-D: Center for Epidemiology Studies-Depression
CI: Confidence interval
IL-6: Interleukin-6
LIFE: Lifestyle Interventions and Independence for Elders
MCI: Mild cognitive impairment
OR: Odds ratio
SD: Standard deviation
SOF: Study of Osteoporotic Fractures
SPPB: Short Physical Performance Battery
