# Supplementary material for: Effect of 24-month physical activity on cognitive frailty and the role of inflammation: the LIFE randomized clinical trial
Source: BMC Med. 2018 Oct 24;16:185. doi: 10.1186/s12916-018-1174-8 (PMC6199791; doi:10.1186/s12916-018-1174-8)
Supplement: Supplementary file 1 — Table S1. Effect of physical activity on cognitive frailty using constrained ordinal logistic regression models. (DOCX 29 kb) [file 12916_2018_1174_MOESM1_ESM.docx]

**Table S1** Effect of physical activity on cognitive frailty using constrained ordinal logistic regression models

|  | **Model 1** | | **Model 2** | | **Model 3** | |
| --- | --- | --- | --- | --- | --- | --- |
|  | OR (95% CI) | *P* Value | OR (95% CI) | *P* Value | OR (95% CI) | *P* Value |
| **Primary analysis** |  |  |  |  |  |  |
| Physical activity vs. health education at 24 months | 0.80 (0.65-0.98) | 0.031 | 0.80 (0.65-0.98) | 0.030 | 0.76 (0.60-0.98) | 0.032 |
| Higher IL-6 vs. lower IL-6 |  | ─ | 1.12 (0.95-1.33) | 0.176 | 1.26 (1.00-1.59) | 0.050 |
| **Secondary analysis** |  |  |  |  |  |  |
| Physical activity vs. health education at 24 months | 0.77 (0.63-0.95) | 0.015 | 0.77 (0.63-0.95) | 0.015 | 0.75 (0.59-0.96) | 0.024 |
| Higher IL-6 vs. lower IL-6 | ─ | ─ | 1.13 (0.96-1.34) | 0.139 | 1.28 (1.01-1.62) | 0.039 |

Abbreviations: OR, odds ratio; CI, confidence interval; IL-6, interleukin-6. The 1,303 participants who had data on either baseline or follow-up cognitive frailty, and baseline IL-6 were included. Constrained ordinal logistic regression was used as described in the Method section. For the primary analysis, ordinal variable 1 was created by assigning 0 for no cognitive frailty (i.e., non-frail without mild cognitive impairment [MCI]), 1 for pre-frail without MCI, 2 for frail without MCI, 3 for non-frail with MCI, 4 for pre-frail with MCI, and 5 for cognitive frailty (i.e., frail with MCI). For the secondary analysis, ordinal variable 2 was created by assigning 2 for non-frail with MCI and 3 for frail without MCI while the other values remained unchanged.

Model 1 adjusted for field center, sex, visit, and intervention by visit interaction.

As the interaction between intervention groups and IL-6 subgroups was not statistically significant (*P* for interaction = 0.170 for primary analysis and 0.276 for secondary analysis), it was not included in Model 2.

Model 3 adjusted for the same covariates as in Model 2 but weighted for the inverse probability of remaining in the study.
