# Supplementary material for: Effect of 24-month physical activity on cognitive frailty and the role of inflammation: the LIFE randomized clinical trial
Source: BMC Med. 2018 Oct 24;16:185. doi: 10.1186/s12916-018-1174-8 (PMC6199791; doi:10.1186/s12916-018-1174-8)
Supplement: Supplementary file 2 — Research investigators for the LIFE Study. (DOCX 15 kb) [file 12916_2018_1174_MOESM2_ESM.docx]

**Research investigators for the LIFE Study**

Administrative Coordinating Center, University of Florida, Gainesville, FL: Marco Pahor, MD – Principal Investigator of the LIFE Study; Jack M. Guralnik, MD, PhD – Co-Investigator of the LIFE Study (University of Maryland School of Medicine, Baltimore, MD); Christiaan Leeuwenburgh, PhD; Connie Caudle; Lauren Crump, MPH; Latonia Holmes; Jocelyn Lee, PhD; Ching-ju Lu, MPH. Data Management, Analysis and Quality Control Center, Wake Forest University, Winston Salem, NC: Michael E. Miller, PhD – DMAQC Principal Investigator; Mark A. Espeland, PhD – DMAQC Co-Investigator; Walter T. Ambrosius, PhD; William Applegate, MD; Daniel P. Beavers, PhD, MS; Robert P. Byington, PhD, MPH, FAHA; Delilah Cook, CCRP; Curt D. Furberg, MD, PhD; Lea N. Harvin, BS; Leora Henkin, MPH, Med; John Hepler, MA; Fang-Chi Hsu, PhD; Laura Lovato, MS; Wesley Roberson, BSBA; Julia Rushing, BSPH, MStat; Scott Rushing, BS; Cynthia L. Stowe, MPM; Michael P. Walkup, MS; Don Hire, BS;

W. Jack Rejeski, PhD; Jeffrey A. Katula, PhD, MA; Peter H. Brubaker, PhD; Shannon L. Mihalko, PhD; Janine M. Jennings, PhD; Shyh-Huei Chen, PhD; June J. Pierce, AB; Haiyeng Chen, PhD. National Institutes of Health, Bethesda, MD: Evan C. Hadley, MD (National Institute on Aging); Sergei Romashkan, MD, PhD (National Institute on Aging); Kushang V. Patel, PhD (National Institute on Aging). National Heart, Lung and Blood Institute, Bethesda, MD: Denise Bonds, MD, MPH. Field Centers: Northwestern University, Chicago, IL: Mary M. McDermott, MD – Field Center Principal Investigator; Bonnie Spring, PhD – Field Center Co-Investigator; Joshua Hauser, MD – Field Center Co-Investigator; Diana Kerwin, MD – Field Center Co-Investigator; Kathryn Domanchuk, BS; Rex Graff, MS; Alvito Rego, MA. Pennington Biomedical Research Center, Baton Rouge, LA: Timothy S. Church, MD, PhD, MPH – Field Center Principal Investigator; Steven N. Blair, PED (University of South Carolina); Valerie H. Myers, PhD; Ron Monce, PA-C; Nathan E. Britt, NP; Melissa Nauta Harris, BS; Ami Parks McGucken, MPA, BS; Ruben Rodarte, MBA, MS, BS; Heidi K. Millet, MPA, BS; Catrine Tudor-Locke, PhD, FACSM; Ben P. Butitta, BS; Sheletta G. Donatto, MS, RD, LDN, CDE; Shannon H. Cocreham, BS. Stanford University, Palo Alto, CA: Abby C. King, PhD – Field Center Principal Investigator; Cynthia M. Castro, PhD; William L. Haskell, PhD Randall S. Stafford, MD, PhD; Leslie A. Pruitt, PhD; Kathy Berra, MSN, NP-C, FAAN; Veronica Yank, MD. Tufts University, Boston, MA: Roger A. Fielding, PhD – Field Center Principal Investigator; Miriam E. Nelson, PhD – Field Center Co-Investigator; Sara C. Folta, PhD – Field Center Co-Investigator; Edward M. Phillips, MD; Christine K. Liu, MD; Erica C. McDavitt, MS;

Kieran F. Reid, PhD, MPH; Dylan R. Kirn, BS; Evan P. Pasha, BS; Won S. Kim, BS; Vince E. Beard, BS; Eleni X. Tsiroyannis, BS; Cynthia Hau, BS, MPH. University of Florida, Gainesville, FL: Todd M. Manini, PhD – Field Center Principal Investigator; Marco Pahor, MD – Field Center Co-Investigator; Stephen D. Anton, PhD; Susan Nayfield, MD; Thomas W. Buford, PhD; Michael Marsiske, PhD; Bhanuprasad D. Sandesara, MD; Jeffrey D. Knaggs, BS; Megan S. Lorow, BS; William C. Marena, MT, CCRC; Irina Korytov, MD; Holly L. Morris, MSN, RN, CCRC (Brooks Rehabilitation Clinical Research Center, Jacksonville, FL); Margo Fitch, PT (Brooks Rehabilitation Clinical Research Center, Jacksonville, FL); Floris F. Singletary, MS, CCC-SLP (Brooks Rehabilitation Clinical Research Center, Jacksonville, FL); Jackie Causer, BSH, RN (Brooks Rehabilitation Clinical Research Center, Jacksonville, FL); Katie A. Radcliff, MA (Brooks Rehabilitation Clinical Research Center, Jacksonville, FL). University of Pittsburgh, Pittsburgh, PA: Anne B. Newman, MD, MPH – Field Center Principal Investigator; Stephanie A. Studenski, MD, MPH – Field Center Co-Investigator; Bret H. Goodpaster, PhD;

Nancy W. Glynn, PhD; Oscar Lopez, MD; Neelesh K. Nadkarni, MD, PhD Kathy Williams, RN, BSEd, MHSA; Mark A. Newman, PhD; George Grove, MS Janet T. Bonk, MPH, RN; Jennifer Rush, MPH; Piera Kost, BA (deceased); Diane G. Ives, MPH. Wake Forest University, Winston Salem, NC: Stephen B. Kritchevsky, Ph.D. – Field Center Principal Investigator; Anthony P. Marsh, PhD – Field Center Co-Investigator; Tina E. Brinkley, PhD Jamehl S. Demons, MD; Kaycee M. Sink, MD, MAS; Kimberly Kennedy, BA, CCRC; Rachel Shertzer-Skinner, MA, CCRC; Abbie Wrights, MS; Rose Fries, RN, CCRC; Deborah Barr, MA, RHEd, CHES.

Yale University, New Haven, CT: Thomas M. Gill, MD – Field Center Principal Investigator;

Robert S. Axtell, PhD, FACSM – Field Center Co-Investigator (Southern Connecticut State University, Exercise Science Department); Susan S. Kashaf, MD, MPH (VA Connecticut Healthcare System); Nathalie de Rekeneire, MD, MS; Joanne M. McGloin, MDiv, MS, MBA; Karen C. Wu, RN; Denise M. Shepard, RN, MBA; Barbara Fennelly, MA, RN; Lynne P. Iannone, MS, CCRP; Raeleen Mautner, PhD; Theresa Sweeney Barnett, MS, APRN; Sean N. Halpin, MA; Matthew J. Brennan, MA; Julie A. Bugaj, MS; Maria A. Zenoni, MS; Bridget M. Mignosa, AS. Cognition Coordinating Center, Wake Forest University, Winston Salem, NC:

Jeff Williamson, MD, MHS – Center Principal Investigator; Kaycee M Sink, MD, MAS – Center Co-Investigator; Hugh C. Hendrie, MB, ChB, DSc (Indiana University); Stephen R. Rapp, PhD;

Joe Verghese, MB, BS (Albert Einstein College of Medicine of Yeshiva University); Nancy Woolard; Mark Espeland, PhD; Janine Jennings, PhD; Valerie K. Wilson, MD. Electrocardiogram Reading Center, University of Florida, Gainesville, FL: Carl J. Pepine MD, MACC; Mario Ariet, PhD; Eileen Handberg, PhD, ARNP; Daniel Deluca, BS; James Hill, MD, MS, FACC; Anita Szady, MD. Spirometry Reading Center, Yale University, New Haven, CT:

Geoffrey L. Chupp, MD; Gail M. Flynn, RCP, CRFT; Thomas M. Gill, MD; John L. Hankinson, PhD (Hankinson Consulting, Inc.); Carlos A. Vaz Fragoso, MD. Cost Effectiveness Analysis Center: Erik J. Groessl, PhD (University of California, San Diego and VA San Diego Healthcare System); Robert M. Kaplan, PhD (Office of Behavioral and Social Sciences Research, National Institutes of Health).
